# Endometriosis: Novel Models, Diagnosis, and Treatment

**DOI:** 10.1155/2014/140413

**Published:** 2014-12-30

**Authors:** Renato Seracchioli, Giulia Montanari, Mohamed Mabrouk, Joseph Nassif

**Affiliations:** ^1^Gynecology & Pathophysiology of Human Reproduction Unit, University of Bologna, Sant'Orsola-Malpighi Hospital, Bologna, Italy; ^2^Department of Gynecology and Obstetrics, Sacred Heart Hospital, Negrar, Verona, Italy; ^3^Obstetrics and Gynecology Department, American University of Beirut Medical Center, Lebanon

Endometriosis remains a cause of significant morbidity in reproductive-aged women resulting in pelvic pain, pelvic masses, and infertility. Endometriosis is defined as the presence of endometrial glands and stroma outside of their normal intrauterine location, most commonly in the dependent portions of the pelvis. Endometriosis is treated with medical therapies, surgery or both. The medical therapies include oral contraceptive pills, progestins, gonadotropin releasing hormone analogues, and danazol. All of these medical therapies induce a hormonal steady state that results in an environment not conducive to the growth of endometriosis. Surgical therapies for endometriosis-associated pain include the removal of endometriotic implants and adhesions with restoration of normal anatomy. Laparoscopy is an effective surgical approach with the goal of excising visible endometriosis. Since endometriosis is a chronic condition, it is not uncommon for recurrences to occur. While endometriosis remains an enigmatic disease, the introduction of new pharmacologic agents and newer endoscopic methods of surgical treatment has facilitated and improved the overall management of this disease.

This special issue contains some papers that refer to molecular and cellular mechanisms of endometriosis physiopathology and some papers that refer to biomarker development for improved early diagnosis and risk of disease, while the other papers refer minimally to invasive treatment for endometriosis and new medical therapies. Finally, there are some manuscripts about prevention of endometriosis and novel models in endometriosis research.

In the paper entitled “ABO and Rhesus Blood Groups and Risk of Endometriosis in a French Caucasian Population of 633 Patients Living in the Same Geographic Area,” B. Borghese et al. found that Rh-negative women have a higher risk of endometriosis; ABO blood group does not influence the risk of endometriosis.

In the paper entitled “Are Mood and Anxiety Disorders and Alexithymia Associated with Endometriosis? A Preliminary Study,” G. Cavaggioni et al. found that some psychopathological aspects, such as psychoemotional distress and alexithymia, are more frequent in women with endometriosis and might amplify pain symptoms in these patients.

In the paper entitled “Antiangiogenesis Therapy of Endometriosis Using PAMAM as a Gene Vector in a Noninvasive Animal Model,” N. Wang et al. found that endostatin-loaded PAMAM inhibits the development of endometriosis through an antiangiogenic mechanism and can be observed through the noninvasive endometriosis mode.

In the paper entitled “Looking for Celiac Disease in Italian Women with Endometriosis: A Case Control Study,” L. Santoro et al evidenced that, in Italian population, an increased prevalence of celiac disease among patients with endometriosis is found, although this trend does not reach the statistical significance.

In the paper entitled “Endometriosis Patients in the Postmenopausal Period: Pre- and Postmenopausal Factors Influencing Postmenopausal Health,” D. Haas et al. suggested that physical fitness and freedom from physical restrictions, a good social environment, and psychological care in both the premenopausal and postmenopausal periods lead to marked improvements in the postmenopausal period with regard to pain, dyspareunia, and influence on sexual life in endometriosis patients.

In the paper entitled “Evaluation of the Usefulness of the MRI Jelly Method for Diagnosing Complete Cul-de-Sac Obliteration,” I. Kikuchi et al. conducted a single-center study to evaluate the usefulness of the magnetic resonance (MR) imaging jelly method for diagnosing endometriosis-associated adhesions in the Pouch of Douglas. The sensitivity and specificity of MR with jelly administration were 95.2% and 88.9%. Opacity produced by the jelly increased the sensitivity and specificity.

In the paper entitled “Medical Treatments for Endometriosis-Associated Pelvic Pain,” G. Zito et al. reviewed the pharmacological agents which have been tested for the treatment of endometriosis-associated pelvic pain. Some of them were ineffective and others proved unfit for clinical use due to significant side effects, while some others seem to be very promising but should be investigated in RCTs. Following the results of the controlled studies available, to date, the first line treatment for endometriosis associated pain is still represented by oral contraceptives used continuously. Progestins represent an acceptable alternative. GnRH analogues may be used as second line treatment, but significant side effects should be taken into account. Other agents such as GnRH-antagonist, aromatase inhibitors, immunomodulators, selective progesterone receptor modulators, and histone deacetylase inhibitors seem to be very promising.

In the paper entitled “Long-Term Outcome after Laparoscopic Bowel Resections for Deep Infiltrating Endometriosis: A Single-Center Experience after 900 Cases,” G. Ruffo et al. confirm that bowel resections for endometriosis are correlated with an acceptable complication rate even at long-term follow-up and that symptoms significantly improve over time, except for rectal bleeding and dysuria.

In the paper entitled “A Deregulated CCN Matricellular Proteins Network in Endometriotic Tissues,” B. Borghese et al. demonstrated that the CCN protein network is deregulated in the ectopic endometrium of women with endometriosis toward a fibrotic process.

In the paper entitled “The Effects of Sunitinib on Endometriosis: An Experimental Rat Model Study” by T. Akman et al., the volume of the endometriotic implants was reduced after sunitinib treatment. Adhesion formation decreased significantly. Therefore, sunitinib treatment seems effective for endometriotic peritoneal lesions. The effects of sunitinib in rat models give hope to improved treatment of human endometriosis to prevent pain symptoms.

In the paper entitled “Endometriosis and Nutrition: Analysis of Food Intake in Patients with Histologically Proven Endometriosis,” B. Kaiseret al. showed that nutrition counseling can help to prevent endometriosis and to maintain the situation following surgery. It may influence the course of endometriosis by reducing the pain and keeping the recurrence rate low.

In the paper entitled “Frequently Misdiagnosed Extrapelvic Sites of Endometriosis: Clinical Cases and Review of the Literature,” E. Fuggetta et al. underlined that extrapelvic endometriosis is a rare condition defined as the presence of endometriotic stroma and glands outside the pelvis and elsewhere in the body. Because of the lower occurrence rate of extrapelvic endometriosis, as well as its unusual sites of involvement, it is often confused with other pathologic conditions. This is the reason why the diagnosis and management are difficult and challenging.

In the paper entitled “Endometriosis: Alternative Methods of Medical Treatment,” A. Tejerizo-García et al. found that hormonal treatments currently available are effective in the relief of pain associated with endometriosis. Among new hormonal drugs, aromatase inhibitors seem to be effective in the relief of pain associated with another treatment in women who did not respond to other treatments. GnRH-antagonist is expected to be as effective as GnRH agonist, but with easier administration (oral). As there is a need to find effective treatments which do not block ovarian function, antiangiogenic factors could be important components of endometriosis therapy in the future.

In the paper entitled “Correlation between Dioxin and Endometriosis: Unraveling the Pathogenesis of the Disease,” O. Triolo et al. aimed to review evidence about the effect of TCDD on eutopic and ectopic endometrium, in order to unravel the machinery behind the dysregulation of immune and hormonal homeostasis caused by this environmental toxicant.

In the paper entitled “Lymphatic Spread in Deep Infiltrating Endometriosis of the Rectosigmoid Bowel,” C. Letzkus and S. Rimbach described a case of deep infiltrating endometriosis of the rectosigmoid, which supports the concept of lymphatic spread from a clinical-pathological point of view by identifying and describing endometriotic implants in a perirectal lymph node and its afferent lymph vessel. The observed case is compared to results of a literature review with regard to the pathogenetic significance of lymphatic spread in endometriosis.

In the paper entitled “An Update of MR Imaging in Endometriosis,” C. van Kuijk et al. aimed to review MR imaging findings of (deep infiltrating) endometriosis in order to explore and summarize experiences in the field of MR imaging in endometriosis. MR imaging in addition to transvaginal ultrasonography (TVS) shows accurate diagnosis of endometriosis and may be predominantly useful for the analysis of patients suspected of deep infiltrating endometriosis (DIE).

In the paperentitled “PJGR Hot-Dog Implant: A New Feasible Rat Model for Surgically Induced Internal and External Endometriosis,” P. G. Ramos et al. studied the possibility of inducing internal or external endometriosis in Wistar rats through a new microsurgical model known as “PJGR Hot-Dog.” This new implant model could enhance our understanding of the mechanisms involved in the development of endometriosis, both internal and external.

